# Adverse events during the titration phase of interferon-beta in remitting-relapsing multiple sclerosis are not predicted by body mass index nor by pharmacodynamic biomarkers

**DOI:** 10.1186/1471-2377-13-82

**Published:** 2013-07-11

**Authors:** Delicias Muñoz, Antonio Escartín, Dolores Dapena, Francisco Coret, Dionisio Fernández-Uría, Domingo Pérez, Bonaventura Casanova, Cristina Guijarro-Castro, Elvira Munteis, María del-Campo Amigo, Robustiano Pego, Carmen Calles, César García-Rey, Nuria Monsalve, David Sánchez-Matienzo

**Affiliations:** 1 Department of Neurology, Hospital Xeral-Cies, Pontevedra, Spain; 2 Department of Neurology, Hospital de la Santa Creu i Sant Pau, Barcelona, Spain; 3 Department of Neurology, Hospital Universitario de Santiago, Santiago de Compostela, A Coruña, Spain; 4 Department of Neurology, Hospital Clínico, Valencia, Spain; 5 Department of Neurology, Hospital de Cabueñes, Gijón, Asturias, Spain; 6 Department of Neurology, Hospital del Bierzo, Ponferrada, León, Spain; 7 Department of Neurology, Hospital Universitario y Politécnico La Fe, Valencia, 46026, Spain; 8 Department of Neurology, Hospital Doce de Octubre, Madrid, Spain; 9 Department of Neurology, Hospital del Mar, Barcelona, Spain; 10 Department of Neurology, Hospital de Pontevedra, Pontevedra, Spain; 11 Department of Neurology, Hospital Lucus Augusti, Lugo, Spain; 12 Department of Neurology, Hospital Universitari Son Espases, Palma de Mallorca, Spain; 13 Medical Adviser, Medical Writing, Madrid, Spain; 14 Medical Department, Merck, S.L., Madrid, Spain

**Keywords:** Interferon β-1a, Body mass index, Biomarkers, β2-microglobulin, OAS, Neopterin, Adverse events

## Abstract

**Background:**

This study aimed to correlate body mass index or biomarkers with the frequency of common adverse events (AEs) with subcutaneous IFN β-1a during treatment titration in patients with relapsing-remitting multiple sclerosis previously naïve to IFN β.

**Methods:**

Eighty-four patients (66.3% females) were followed up during 8 weeks, 25.3% were overweight and 14.5% were obese.

**Results:**

Biomarkers steadily increased during all study period by 45.3% for β2-microglobulin, 262.8% for olygoadenylate synthetase-1, and 92.8% for neopterin. Overall AE reporting did not vary with the dose or treatment duration.

**Conclusions:**

BMI was not predictive of increased risk for AEs. Biomarkers did not discriminate on the frequency of any AE either.

## Background

More than one decade ago, recombinant human interferon β (IFN β) was the first approved disease modifying drug, and still remains as a standard first-line therapy for relapsing-remitting multiple sclerosis (RRMS).

When IFN β binds its receptors on the cell membrane, it induces the secretion of proteins with antiviral, antiproliferative and immunomodulator activities. β2-microglobulin (β2M), 2’-5’-oligoadenylate synthethase-1 (OAS1) and neopterin (Np) are among the best known pharmacodynamic biomarkers often used to assess the IFN β biological activity [1-4] and these are usually inversely correlated with the presence of anti-interferon neutralizing antibodies [5-7].

The 2,5-oligoadenylate synthetase 1 (OAS 1) system is an IFN-induced antiviral pathway, which confers protective and antiproliferative properties. When activated, OAS proteins catalyse the polymerization of ATP into 2'-5'-linked oligomers. Some polymorphisms of OAS1 gene may confer susceptibility to develop multiple sclerosis [8].

β2-microglobulin (β2MG) is associated with class I antigens of the major histocompatibility complex on the surface of lymphocytes and is considered to be a marker for disease activity in some immune and neoplastic disorders. Niezgoda et al. found a significant decrease in cerebrospinal fluid and serum β2MG levels in MS patients after cladribine treatment, associated with a slight but significant clinical improvement measured by EDSS [9]. However, Bagnato et al. followed the evolution of serum β2MG in untreated patients and failed to show any relationship between this marker and disease activity [4].

Neopterin (Np) is synthesized by macrophages upon stimulation with interferon and it is indicative of a pro-inflammatory immune status. Np serves as a marker of cellular immune system activation under the control of T helper cells type 1. Elevated Np concentrations are among the best predictors of adverse outcome in patients with HIV infection, in cardiovascular disease and in various types of cancer [10]. Most studies however have not found a clear correlation with multiple sclerosis progression [3,11].

Patients with MS tend to be more overweight than the general population [12] .Obesity seems to contribute to a delay in the diagnosis of MS through a still undetermined mechanism [13].

IFN-beta-1-a-induced immunomodulation in vivo depends on the administration schedule being 2–3 times greater the effect when a same weekly dose is divided in three injections [14].

Indicators of treatment response or drug-related AEs, either non-modifiable pharmacogenetic indicators like polymorphisms of genes or pharmacodynamic markers [15] might contribute to identify patients who can better benefit from treatment.

Recently, a study showed differences between responders and non responders in the genes associated with ion channels and signal transduction pathways. This study also suggests that genetic variants in heparan sulfate proteoglycan genes may be of clinical interest in MS as predictors of the response to therapy [16] .So far, no study has addressed a correlation of either body mass index (BMI) or IFN β biomarkers with the frequency of common AEs, which could help develop a more individualized IFN β therapy. The aim of the present clinical trial was to address whether variations of BMI or pharmacodynamic biomarkers are associated to different AE profiles during the titration phase of IFN β-naïve patients with RRMS.

## Methods

This interventional open-label, single arm trial (EudraCT Number 2006-000606-23) was performed in 12 hospitals in Spain from July 2008 to June 2010, and was approved by all the Institutional Review Boards prior to initiation. All patients signed an informed consent form before enrollment, and were included in the study if aged between 18 and 60 years, diagnosed with RRMS, had suffered from one or more relapses within the previous 12 months, had an Expanded Disability Status Scale (EDSS) score < 5.5, and had not been previously treated with any IFN β or any other disease modifying drug (DMD). Exclusion criteria included progressive forms of MS, current or past liver abnormal function, leukocytopenia during the month prior to the start of the study, and presence of any other systemic disease.

The study treatment was IFN β-1a (Rebif^®^, Merck Serono), administered subcutaneously three times per week, starting with a dose of 8.8 μg for 2 weeks, then increasing up to 22 μg for other 2 weeks, and finally from day 28 on, at the targeted dose of 44 μg. The study end was after 56 days, following which patients were treated based on normal physician’s practice. On days 1, 14, 28 and 56 blood samples were taken before the next administration of the study drug to perform a complete blood cell count, analysis of liver and renal function, and also determination of the three MS biomarkers: β2M was measured by chemiluminescence (Immulite2000, Siemens), Np by ELISA (DRG International) and OAS1 by RIA (Izasa). Adverse event logs were documented at each visit.

At the same time serum samples were stored frozen at -70C until shipped to a central laboratory for the determination of the three biomarkers β2 microglobulin, neopterin and 2’-5’ oligoadenylate synthetase. β2 microglobulin was measured by chemiluminescence with reagents provided by Siemens. Neopterin was assessed by enzyme-linked immunosorbent assay (ELISA) using DRG International reagents. 2’-5’ oligoadenylate synthetase was determined by means of radioimmunoassay with reagents from Izasa. An adverse event log was completed at each visit. The Student t-test for related and unrelated samples was used to compare means as appropriate. The correlation between BMI, biomarkers and lab results was also assessed using the Pearson or Spearman coefficients (for variables of normal or non parametric distribution according to the Kolmogorov-Smirnov test). The significance level was set at 0.05.

The means of the biochemical, hematological and biomarkers values will be compared to the baseline with a Student-t test for related samples so as to assess the evolution of these variables. The change in the biochemical, hematological and biomarkers values was also compared to the BMI by means of a scattergram and the Pearson (for variables of normal distribution) or Spearman (for non parametric variables) coefficients. The BMI mean of patients with and without headache, influenza-like syndrome and local toxicity with was compared with Student-t test for unrelated samples.

Likewise, the relationship between both the basal value of the three biomarkers and their change over time, among patient with or without adverse events was assessed with the Student-t test for unrelated samples. As for the relation between biomarkers and biochemical and hematological variables, their correlation by means of the Pearson or Spearman coefficients was also explored.

The primary study endpoint aimed to correlate the level of the three biomarkers, adjusted by BMI, and their association with the frequency of AEs. As a secondary endpoint, we aimed to further study these three biomarkers, adjusted by BMI, with the seriousness of AEs.

## Results and discussion

A total of 84 patients were enrolled in this study. One patient was lost to follow up after first visit, and 83 patients completed the full study. Demographic characteristics (mean and standard deviation (SD)) of study population are summarized as follows: weight 70.7 kg (17.0), height 166.3 cm (9.7), age 36.6 years (9.2), time from diagnosis to treatment 16.4 days (4.0), baseline EDSS 1.7 (1.0), 12 months before study EDSS (only available for 33 patients) 0.9 (1.1) and BMI 25.5 kg/m [2] (5.5). As for gender, female proportion was 66.3%. 60.2% of patients had normal BMI (< 25 kg/m2), 25.3% were overweight (≥25 y < 30 kg/m2) and 14.5% were obese (≥ 30 kg/m2). The proportion of relapses in the previous 12 months before the study was 1 in 59.0% of patients, 2 in 32.3% and 3 or more, in 9.6%.

Baseline and final 56-day mean values of the different continuous variables studied are shown in Table 1.

**Table 1:** Basal and final means of biochemical, hematological and biomarker variables

**Variable**	**Basal mean (SD) [No. of Valid cases]**	**Final mean (SD) [No. of Valid cases]**	p*	**Pearson correlation (BMI)**	**Spearman correlation (BMI)**
Haemoglobin (g/dL)	13.94 (1.16) [81]	13.64 (1.28) [81]	NS	-0.015	0.095
Hematocrit (%)	41.48 (3.50) [81]	40.56 (3.61) [81]	NS	-0.095	0.020
Blood sedimentation rate (mm)	12.31 (10.89) [58]	14.49 (11.65) [71]	NS	0.112	0.067
Leukocytes (1000/mm [3])	8.25 (3.14) [83]	5.67 (1.67) [81]	<0.01	-0.081	0.056
Granulocytes (1000/mm [3])	5.29 (2.88) [82]	3.20 (1.22) [81]	<0.01	-0.127	0.014
Lymphocytes (1000/mm [3])	2.32 (1.10) [83]	1.76 (0.61) [81]	<0.01	-0.060	0.106
Platelet count (1000/mm [3]	275.59 (81.21) [83]	211.39 (63.98) [81]	<0.01	0.078	0.158
Creatinine	0.78 (0.17) [82]	0.74 (0.15) [81]	0.02	-0.117	-0.091
sGOT (ALT)	17.50 (5.99) [80]	34.11 (39.75) [81]	<0.01	-0.091	-0.149
sGPT (AST)	23.34 (15.66) [80]	53.56 (98.87) [81]	<0.01	-0.149	-0.178
GGT	19.18 (11.23) [68]	36.34 (36.93) [73]	<0.01	-0.102	-0.008
Alkaline phosphatase	95.54 (46.27) [67]	101.24 (48.73) [75]	NS	-0.037	-0148
Bilirubin	0.60 (.36) [78]	0.53 (0.30) [81]	0.025	0.084	0.049
β2M	1.27 (0.23) [83]	1.84 (0.26) [83]	<0.01	-0.189	-0.259
OAS1	50.65 (45.04) [79]	183.75 (99.52) [83]	<0.01	0.003	-0.043
Np	1.46 (0.68) [83]	2.81 (0.81) [83]	<0.01	0.034	-0.043

* Paired samples test for difference of means (2-tailed).

Biomarkers also followed a significant (p < 0.01) and steady increase at 56 days after treatment compared with baseline values: β2M increased by 45.3%, OAS1 by 262.8% and Np by 92.8%.

As most common AEs, influenza-like symptoms were reported in 28 subjects (33.7%), headache in 15 (18.1%) and local reactions at the injection site in 11 subjects (13.3%). With regard to blood analysis, there were 8 cases of abnormal liver enzymes (9.6%), 4 cases of leukocytopenia (4.8%) and one case of thrombocytopenia (1.2%). Three (3.6%) patients were withdrawn from the study, 2 of them at second visit on day 14, due to severe chills and suicide thinking, respectively, and the third patient at third visit on day 28 due to a severe increase of transaminases.

Frequencies of AEs did not increase as the study progressed, despite dose escalation. The overall proportion of subjects reporting any AE was similar at each study visit (39.5% on day 14, 42% on day 28, and 42% on day 56).

No association between mean BMI and frequency of total or any specific AE was established. Similarly, no strong associations were found between BMI and variations of studied biomarkers (all correlations were under ±0,03). Additionally, no significant differences between either the mean baseline value or the variations of biomarkers were found to be associated to patients reporting any AE vs. those non reporting AEs (see Table 1).

## Conclusion

In conclusion, we found that BMI and pharmacodynamic biomarkers were not predictive of an increased frequency of AEs.

To our knowledge, these are the first results establishing a lack of correlation between BMI or biomarkers with frequency of AEs for IFN β-1a during the first two months of treatment. Therefore, an individualized approach should not be based on BMI or biomarkers from a safety perspective.

## Competing interests

This study was sponsored by Merck S.L Spain, an affiliate of Merck KGaA, Darmstadt, Germany. Delicias Muñoz, Received honoraria for scientific advice and speaking from de Biogen Idec, Merck Serono, Novartis, Bayer, Teva and Gemzime Sanofi Aventis. Antonio Escartín, Research fee from Bayer-Schering, Merck-Serono, Novartis, Teva and Biogen. Dolores Dapena, Fee for patient recruitment. Francisco Coret, Speaking honoraria, travel expenses for scientific meetings, or has participated in advisory boards of clinical trials in the past years, with the following companies: Biogen Idec, EMD Merck Serono, Novartis, and Teva Pharmaceuticals. Dionisio Fernández-Uría, Speaking honoraria, fee for patient recruitement and assistance for congress attendance from Merck-Serono. Domingo Pérez, Participated in studies carried out by Schering and Biogen. Bonaventura Casanova, Honoraria from Merck, Sanofi, Teva, Roche and Novartis. Cristina Guijarro, Received research funding by Merck-Serono. Actually, Merck-Serono employee. Elvira Munteis, Payment of travel expenses, accommodation and congress attendance. Merk-Serono and Teva-Sanofi: Speaking honoraria., Biogen: Fee for patient recruitment., Novartis, Merk-Serono: Funds for research. Biogen,Bayer, Merk-Serono, Teva-Sanofi,Novartis: Sponsoring contribution for educational programs. Biogen, Bayer, Merk-Serono, Teva-Sanofi, Novartis: María del Campo Amigo. Robustiano Pego, No conflict of interest. Carmen Calles, Fee for patient recruitment, Received speaking honoraria from Novartis, Sanofi and Biogen. César García-Rey, Contracted out by Merck to arrange this manuscript. Nuria Monsalve, Merck-Serono employee. David Sánchez-Matienzo, Merck-Serono employee.

## Authors’ contributions

This study was sponsored by Merck S.L Spain, an affiliate of Merck KGaA, Darmstadt, Germany. DM, AE, DD, FC, DFU, DP, CGC, EM, MCA, RP and CC received fee for patient recruitment. All of them contributed in the analysis and interpretation of data, were involved in drafting the manuscript, revising it critically and in the acquisition of data. CGR contributed to this manuscript as a medical writer. The funding body contribute helping the authors in the study design, collection analysis, interpretation of data and in the writing of the manuscript. All the authors contribute in the study design, in the collection, analysis, and interpretation of data, in the writing of the manuscript and in the decision to submit the manuscript for publication. All authors read and approved the final manuscript.

## Pre-publication history

The pre-publication history for this paper can be accessed here:

http://www.biomedcentral.com/1471-2377/13/82/prepub
